# Non-invasive assessment of liver fibrosis in autoimmune hepatitis: Diagnostic value of liver magnetic resonance parametric mapping including extracellular volume fraction

**DOI:** 10.1007/s00261-020-02822-x

**Published:** 2020-10-19

**Authors:** Narine Mesropyan, Patrick Kupczyk, Leona Dold, Tobias J. Weismüller, Alois M. Sprinkart, Burkhart Mädler, Claus C. Pieper, Daniel Kuetting, Christian P. Strassburg, Ulrike Attenberger, Julian A. Luetkens

**Affiliations:** 1 Department of Diagnostic and Interventional Radiology, University Hospital Bonn, Venusberg-Campus 1, 53127 Bonn, Germany; 2 Quantitative Imaging Lab Bonn (QILaB), Venusberg-Campus 1, 53127 Bonn, Germany; 3 Department of Internal Medicine I, University Hospital Bonn, Venusberg-Campus 1, 53127 Bonn, Germany; 4 Philips GmbH Germany, Roentgenstrasse 22, 22335 Hamburg, Germany

**Keywords:** Autoimmune hepatitis, Magnetic resonance imaging, Magnetic resonance elastography

## Abstract

**Purpose:**

Autoimmune hepatitis (AIH) is an immune-mediated chronic liver disease that leads to severe fibrosis and cirrhosis. The aim of this study was to determine the diagnostic value of T1 and T2 mapping as well as extracellular volume fraction (ECV) for non-invasive assessment of liver fibrosis in AIH patients.

**Methods:**

In this prospective study, 27 patients (age range: 19–77 years) with AIH underwent liver MRI. T1 and T2 relaxation times as well as ECV were quantified by mapping techniques. The presence of significant fibrosis (≥ F2) was defined as magnetic resonance elastography (MRE)-based liver stiffness ≥ 3.66 kPa. MRE was used as reference standard, against which the diagnostic performance of MRI-derived mapping parameters was tested. Diagnostic performance was compared by utilizing receiver-operating characteristic (ROC) analysis.

**Results:**

MRE-based liver stiffness correlated with both, hepatic native T1 (*r* = 0.69; *P* < 0.001) as well as ECV (*r* = 0.80; *P* < 0.001). For the assessment of significant fibrosis, ECV yielded a sensitivity of 85.7% (95% confidence interval (CI): 60.1–96.0%) and a specificity of 84.6% (CI 60.1–96.0%); hepatic native T1 yielded a sensitivity of 85.7% (CI 60.1–96.0%); and a specificity of 76.9% (CI 49.7–91.8%). Diagnostic performance of hepatic ECV (area under the curve (AUC): 0.885), native hepatic T1 (AUC: 0.846) for assessment of significant fibrosis was similar compared to clinical fibrosis scores (APRI (AUC: 0.852), FIB-4 (AUC: 0.758), and AAR (0.654) (*P* > 0.05 for each comparison)).

**Conclusion:**

Quantitative mapping parameters such as T1 and ECV can identify significant fibrosis in AIH patients. Future studies are needed to explore the value of parametric mapping for the evaluation of different disease stages.

## Introduction

Autoimmune hepatitis (AIH) is an immune-mediated chronic liver disease that may lead to severe liver fibrosis and cirrhosis. AIH is relatively rare and predominantly affects females [1]. According to current guidelines, liver biopsy is recommended in patients with AIH to establish diagnosis, evaluate the presence of fibrosis, and make further treatment decision [2]. However, despite being considered the gold standard for diagnosis, liver biopsy in AIH patients has clear disadvantages, which include high clinical expertise, high intra- and interobserver variability, risk of severe periprocedural complications, and high cost. Also, serial liver biopsies are not practical for long-term monitoring of a patient's treatment response. As fibrosis detection and staging are important for treatment decisions and prognosis estimation reliably non-invasive measurements are needed, in these patients [1].

Magnetic resonance elastography (MRE) is currently regarded the most accurate non-invasive technique for the detection and staging of liver fibrosis [3–5]. Several studies have demonstrated that the diagnostic performance of MRE in this role is superior to that of transient elastography (TE) [4, 6]. In particular, MRE is notable for its ability to accurately diagnose mild fibrosis, which is difficult by TE [7]. However, also MRE has its drawbacks due to high technical failures rate, i.e., in patients with severe ascites or iron overload [6, 8].

The concept of evaluating the T1 relaxation times in differentiating normal from diseased livers was introduced in the 1980s [9, 10]. Hepatic inflammation and fibrosis are believed to increase the T1 relaxation time of liver parenchyma due to an increase in extracellular matrix and water and protein concentration [11]. T1 mapping can depict even small variations of T1 within a tissue and has been used in cardiac imaging to detect myocardial edema, iron overload, myocardial infarcts, and scarring [12]. Furthermore, T1 relaxation times can also be measured before and after the administration of an extracellular contrast agent, which allows the additional calculation of the extracellular volume (ECV). ECV is a measure of the extracellular space and represents the tissue volume, which is not taken by cells [13]. ECV values are calculated from the change in relaxation rate (R1 = 1/T1) of blood and parenchyma corrected for the hematocrit [14, 15]. Similarly, T2 mapping has been reported to be useful in measuring hepatic fibrosis and potentially in differentiating between different stages of liver fibrosis in human and animal studies [15, 16, 22, 23]. Prolonged T2 relaxation times in regions of fibrosis are potentially attributable to the coexistent inflammation and high water content of the advanced fibrosis. There have been several studies showing correlations between hepatic T1, T2, and ECV with liver fibrosis in both animal and human models [15–21].

Therefore, the purpose of our explorative prospective study was to evaluate the diagnostic utility of different quantitative parametric magnetic resonance imaging (MRI) parameters (T1, T2, and ECV) to diagnose liver fibrosis in patients with AIH using MRE-based liver stiffness as a reference standard.

## Material and methods

The institutional review board approved this prospective study, and all study participants provided written informed consent prior to MRI examination. From June 2019 to March 2020, patients with AIH diagnosis were consequently included in this study. Diagnosis of AIH was based on diagnostic criteria of AIH, established by the International Autoimmune Hepatitis Group (IAIHG) [24]. Also patients with overlap syndromes, which implies that the predominant disease is AIH and that the concurrent cholestatic features are background components [25], were included.

All patients included in the study had no acute exacerbation at the time of MRI examination based on clinical and laboratory findings with a good response to immunosuppressive therapy. Model for end-stage liver disease (MELD) was analyzed, and laboratory markers were retrieved from the institutional medical information system. Also, non-invasive scoring systems based on laboratory tests for assessment of liver fibrosis (aspartate aminotransferase-to-platelet ratio index (ARPI), fibrosis index based on the 4 factor (FIB-4), and aspartate aminotransferase and alanine aminotransferase ratio (AST/ALT ratio (de-Ritis)) were calculated as previously described [26–28].

### Multiparametric magnetic resonance imaging

All imaging was performed on a clinical whole-body 1.5 T MRI system (Ingenia, Philips Healthcare) equipped with 32-channel abdominal coil with digital interface for signal reception. Besides morphological sequences, patients underwent MRE and parametric mapping of the liver.

Liver MRE was implemented by 2D gradient-recalled echo to acquire liver elasticity maps with motion-encoding gradients (MEGs). Sequence parameters were as follows: time of repetition (TR) 50 ms, time of echo (TE) 20 ms, flip angle (FA) 20°, parallel imaging factor 2.3, active driver frequency 60 Hz, active driver power 100%, acquired voxel size 1.5 × 4.74 × 10 mm, reconstructed voxel size 1.17 × 1.17 x 10 mm, scan duration/breath hold 15.3 s, and 3 slices. The system configuration was based on a pneumatically powered active wave driver and a tube-connected and strap-secured passive driver placed over the right liver lobe. Generated shear waves at a fixed vibration frequency were coursing through the liver and created tissue displacements, which could be detected to generate magnitude and phase images. Phase shift of magnetic resonance signal was measured at four different phase offsets over one cycle of motion. Further analysis by integrated software (MR elastography View, Philips Healthcare) allowed the creation of a quantitative elastogram (liver stiffness map). For hepatic T1 mapping a heart rate-independent 10-(2)-7-(2)-5-(2)-3-(2), modified Look-locker inversion recovery (MOLLI) acquisition scheme [29] with internal triggering was implemented. The following technical parameters were applied: TR 1.92 ms, TE 0.84 ms, FA 20°, parallel imaging factor 2, acquired voxel size 1.98 × 2.45 × 10 mm, reconstructed voxel size 1.13 × 1.13 × 10 mm, and scan duration/breath hold 14.0 s. Post-contrast T1 maps using the same imaging technique were performed 10 min after contrast injection in the same positions as pre-contrast examinations. For contrast enhancement, the extracellular contrast agent Gadobutrol (1.0 mmol/ml solution with 0.1 mmol per kilogram of body weight, Gadovist, Bayer Healthcare Pharmaceuticals) was injected as a bolus at a rate of 1.5 ml/s and followed by a 10 ml saline flush. For hepatic T2 mapping, a six-echo gradient spin echo sequence (GraSE) was used [30], and scan parameters: TR 450 ms, inter-echo spacing 16 ms, FA 90°, parallel imaging factor 2.5, acquired voxel size 1.98 × 2.01 × 10 mm, reconstructed voxel size 0.88 × 0.88 × 10 mm, scan duration/breath h hold 15/3 × 5 s. T1 and T2 mapping were performed in transversal views covering liver parenchyma at the level of the portal bifurcation. T1 and T2 relaxation maps were reconstructed at the scanner console. Maps of proton density fat fraction (PDFF) and T2* were achieved with a six-echo 3D gradient-echo sequence (mDixon Quant, Philips Healthcare).The following parameters were applied: TR 7.8 ms, TE 1.1 ms, FA 5°, parallel imaging factor 2, acquired voxel size 1.99 × 1.99 × 6 mm, reconstructed voxel size 0.99 × 0.99 × 3 mm, scan duration/breath hold 15.0 s.

### Image analysis

Image analyses were performed by an experienced board-certified radiologist (J.A.L, 8 years of experience in abdominal MRI), blinded for the clinical information. For the assessment of T1 and T2 relaxation times and PDFF, three representative round regions of interest (ROIs) (minimum of one cm^2^) were drawn centrally in three hepatic segments (segments 2, 4a, and 7), and mean relaxation times were calculated [31]. T1 values of the blood pool were obtained from the abdominal aorta on the transversal maps. ECV values were normalized for hematocrit and calculated with regions of interest from pre- and post-contrast T1 values using the following equation [32]: ECV = (1 − hematocrit)*(1/T1 parenchyma post-contrast − 1/T1 parenchyma pre-contrast)/(1/T1 aortic post-contrast − 1/T1 aortic pre-contrast). Blood hematocrit levels were determined on the day of examination. Liver tissue stiffness values were derived from stiffness confidence map by drawing the largest possible freehand ROIs (minimum of one cm^2^) in three different representative regions of the liver. All patients were divided into two groups, without (< fibrosis stage (F) 2) and with significant fibrosis (≥ F2) according to the MRE-based liver stiffness. According to the literature, a cut-off of 3.66 kPa was chosen to differentiate between patients without and with significant liver fibrosis. The cut-off values for F2, F3, and F4 were 3.66, 4.11, and 4.71 kPa, respectively[4, 33].

### Statistical analysis

Statistical analysis was performed using SPSS Statistics (Version 25, IBM) and MedCalc (Version 19.1.3, MedCalc Software). Patient characteristics are presented as mean ± standard deviation or as absolute frequency. Continuous variables between two groups were compared using Student *t* test. Dichotomous variables were compared using the χ2 test (with the cell count greater than five) and Fisher test (with a cell count less than or equal to five). The bivariate Pearson correlation coefficient (*r*) was used for a correlation analyses. Receiver operating characteristic analysis (ROC) was performed to calculate areas under the curve (AUC). AUCs were compared using the method proposed by DeLong et al. [34]. Sensitivity, specificity, and accuracy were calculated. MRE-based liver stiffness was the reference standard against which the diagnostic performance of MRI-derived mapping parameters of liver was tested. A *P* value < 0.05 was considered statistically significant.

## Results

### Cohort characteristics

A total of 27 patients with AIH diagnosis were included in this study. 9/27 (33.3%) patients had an overlap syndrome. At the time of MRI examination, 13/27 (48.1%) patients received immunosuppressive therapy with budesonide alone; 8/27 (29.6%) patients received a combination of budesonide with azathioprine; and 3/27 (11.1%) patients received immunosuppressive therapy with azathioprine alone. There were also 3/27 (11.1%) patients, who received no therapy at the time of MRI examination. 13/27 (48.2%) patients had no or not significant (< F2), and 14/27 (51.8%) had significant (≥ F2) fibrosis. 1/14 (7.1%), 4/14 (28.6%), and 9/14 (64.3%) patients had fibrosis stages F2, F3, and F4, respectively. The mean age of patients with no significant fibrosis according to the MRE was 46.6 ± 18.6 years (range: 20–74 years), with significant fibrosis 42.6 ± 18.6 years (range: 19–77 years). The mean body mass index (BMI) in patients with no significant fibrosis was 26.9 ± 4.3 kg/m^2^, in patients with significant fibrosis 23.5 ± 2.9 kg/m^2^ (*P* = 0.047). Age and sex did not differ in both groups (*P* > 0.05). No significant differences were found between clinical fibrosis scores APRI and AST/ALT ratio in both groups (*P* > 0.05). FIB-4 was higher in the group with significant fibrosis (≥ F2) compared to the group with no significant fibrosis (< F2) (*P* = 0.006). Clinical characteristics are summarized in Table 1.

**Table 1:** Clinical characteristics of patients without significant fibrosis (< F2) and with significant fibrosis (≥ F2)

Variable	Patients with AIH and no significant fibrosis (< F2, n = 13)	Patients with AIH and significant fibrosis (≥ F2, n = 14)	*P* value
Age (years)	46.6 ± 18.6	42.6 ± 18.6	0.585
Body mass index (kg/m^2^)	26.9 ± 4.3	23.5 ± 2.9	0.047
Sex			0.385
Male	2 (15.4%)	5 (35.7%)	
Female	11 (84.6%)	9 (64.3%)	
Hematocrit level (%)	39.9 ± 6.8	40.2 ± 2.2	0.911
MELD	7.4 ± 2.8	7.9 ± 3.1	0.666
Bilirubin (mg/dl)	0.56 ± 0.49	1.27 ± 0.78	0.011
ALT (U/l)	42.3 ± 35.7	145.6 ± 188.2	0.063
AST (U/l)	30.3 ± 16.5	90.6 ± 97.1	0.045
GGT (U/l)	93.5 ± 145.1	171.7 ± 189.9	0.243
Platelets cells × 10^9^/l	291.3 ± 81.5	178.8 ± 100.8	0.003
C-reactive protein level (mg/l)	7.2 ± 5.3	5.1 ± 8.8	0.539
AP (U/l)	98.9 ± 75.6	129.2 ± 127.3	0.468
Creatinine (mg/dl)	0.71 ± 0.16	0.79 ± 0.21	0.315
Albumin (g/l)	41.3 ± 4.0	42.3 ± 6.3	0.717
International normalized ratio	1.12 ± 0.35	1.08 ± 0.08	0.708
ASL/ALT (de-Ritis)	1.07 ± 0.49	0.85 ± 0.35	0.183
FIB-4	1.03 ± 0.50	2.26 ± 1.38	0.006
APRI	0.58 ± 1.02	1.32 ± 1.08	0.084

Continuous data are means ± standard deviations. Nominal data are absolute frequencies with percentages in parentheses

*MELD* score model of end-stage liver disease, *ALT* alanine aminotransferase, *AST* aspartate aminotransferase, *AP* alkaline phosphatase, *GGT* gamma-glutamyltransferase, *APRI* aspartate aminotransferase-to-platelet ratio index, *FIB-4* Fibrosis-4-Score, *ASL/ALT (de-Ritis)* De-Ritis-Quotient

### MRI results

**Fig. 1:**
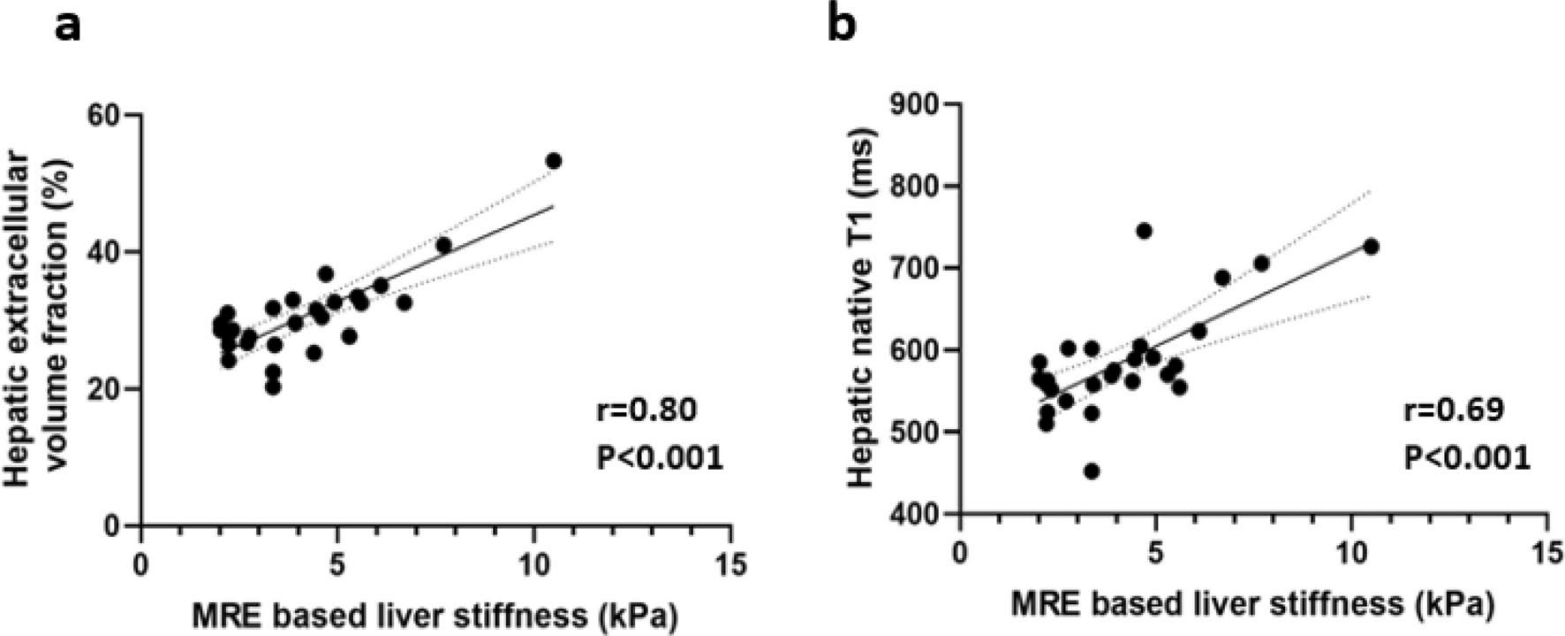
Scatter plots shows correlations between magnetic resonance elastography (MRE)-based liver stiffness and hepatic extracellular volume fraction (**a**) and hepatic native T1 (**b**). Regression line is given with 95% confidence interval

**Fig. 2:**
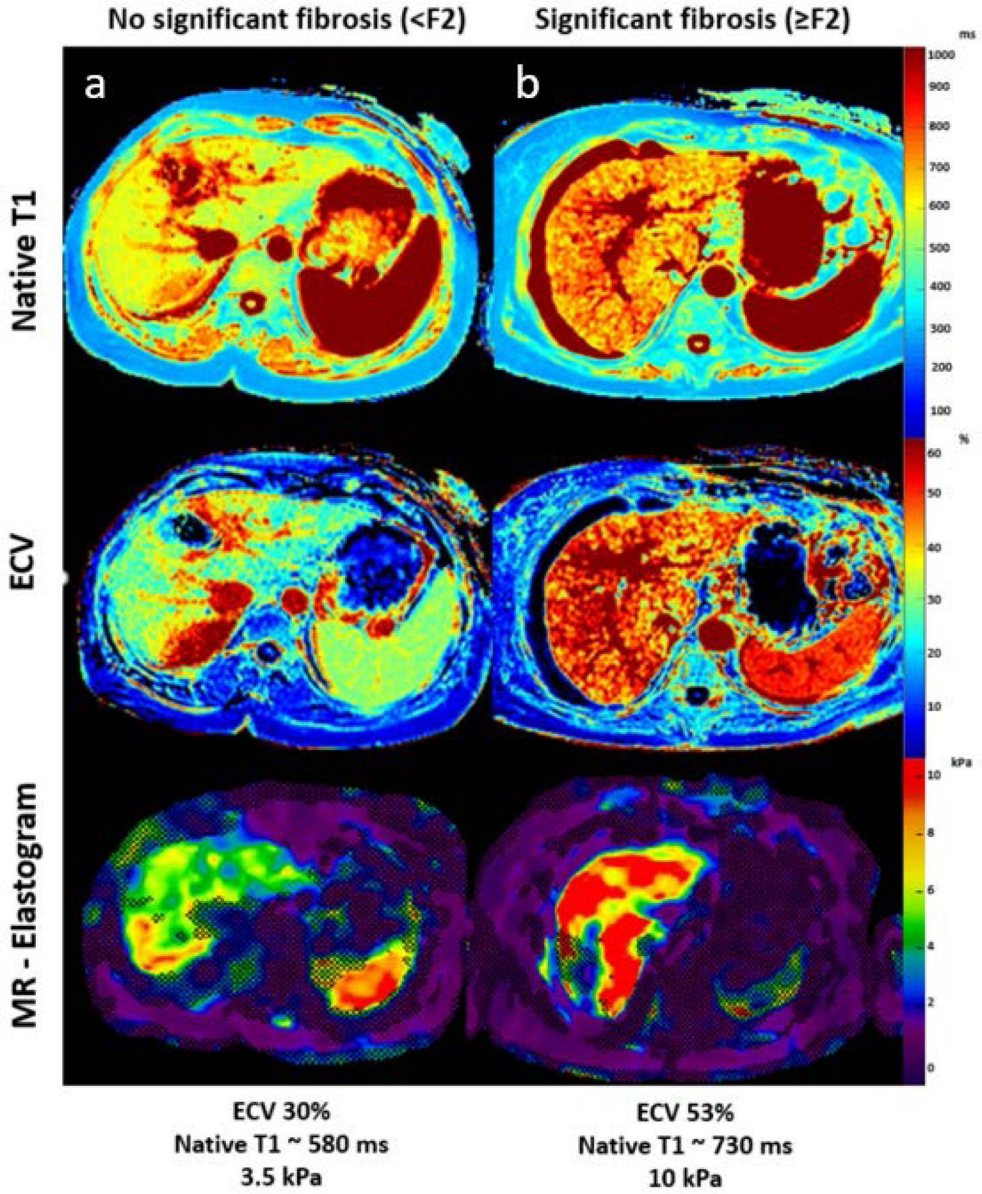
Representative images of hepatic native T1 and extracellular volume (ECV) maps and magnetic resonance elastogram (MRE) from patients with no significant fibrosis (< F2, **a**) and patient with significant fibrosis (≥ F2, **b**). *ECV* extracellular volume fraction, *F* fibrosis stage

Compared to patients with AIH and no significant fibrosis (< F2), patients with significant fibrosis (≥ F2) had markedly increased hepatic native T1 relaxations times (548.8 ± 40.7 ms vs. 620.3 ± 66.3 ms; *P* = 0.003) and hepatic ECV values (27.1 ± 3.2% vs. 38.7 ± 18.9%; *P* = 0.039). There were no significant differences in hepatic T2 relaxation times between both groups (50.7 ± 4.2 ms vs. 50.5 ± 6.5 ms; *P* = 0.920). Also, no significant difference in fat fraction was present in both groups (5.3 ± 4.6% vs. 3.2 ± 1.5%, *P* = 0.135). MRE-based liver stiffness and hepatic parametric MRI results are given in Table 2. Furthermore, we found a strong correlation between MRE-based liver stiffness and hepatic native T1 (*r* = 0.69, *P* < 0.001) as well as hepatic ECV (*r* = 0.80, *P* < 0.001, see also Fig. 1). There were also significant correlations between clinical fibrosis scores such as FIB-4 and APRI and hepatic native T1 (for both scores, *r* = 0.49, *P* < 0.05). Also, hepatic ECV showed a significant correlation with FIB-4 score (*r* = 0.39, *P* = 0.04). We found no correlations between hepatic T2 and MRE-based liver stiffness as well as clinical fibrosis scores (FIB-4 and AST/ALT Ratio). A correlation matrix is given in Table 3. Representative images from patients with and without significant fibrosis are given in Fig. 2.

**Table 2:** Hepatic magnetic resonance elastography characteristics of patients without (< F2) and with significant fibrosis (≥ F2)

Variable	Patients with AIH and no significant fibrosis (< F2, n = 13)	Patients with AIH and significant fibrosis (≥ F2, n = 14)	*P* value
MRE-based liver stiffness (kPa)	2.6 ± 0.6	5.6 ± 1.8	< 0.001
Hepatic native T1 relaxation time (ms)	548.8 ± 40.7	620.3 ± 66.3	0.003
Hepatic extracellular volume fraction (%)	27.1 ± 3.2	38.7 ± 18.9	0.039
Hepatic T2 relaxation time (ms)	50.7 ± 4.2	50.5 ± 6.5	0.920
Hepatic T2* relaxation time (ms)	31.1 ± 5.2	32.5 ± 5.9	0.537
Proton density fat fraction (%)	5.3 ± 4.6	3.2 ± 1.5	0.135

Continuous data are means ± standard deviations

**Table 3:** Correlation matrix for quantitative MRI parameters and clinical fibrosis scores

Variable	Hepatic native T1		Hepatic T2		Hepatic ECV	
	*r* value	*P* value	*r* value	*P* value	*r* value	*P* value
MRE-based liver stiffness	0.69	< 0.001	0.03	0.903	0.80	< 0.001
FIB-4	0.49	0.010	− 0.09	0.625	0.39	0.04
APRI	0.49	0.009	0.42	0.031	0.23	0.25
AST/ALT ratio (de-Ritis)	− 0.06	0.775	− 0.23	0,260	0.02	0.92

*ECV* extracellular volume fraction, *MRE* magnetic resonance elastography, *FIB-4* Fibrosis-4-Score, *ASL/ALT ratio (de-Ritis)* De-Ritis-Quotient, *APRI* aspartate aminotransferase-to-platelet ratio index

### Diagnostic performance of parametric mapping parameters

**Fig. 3:**
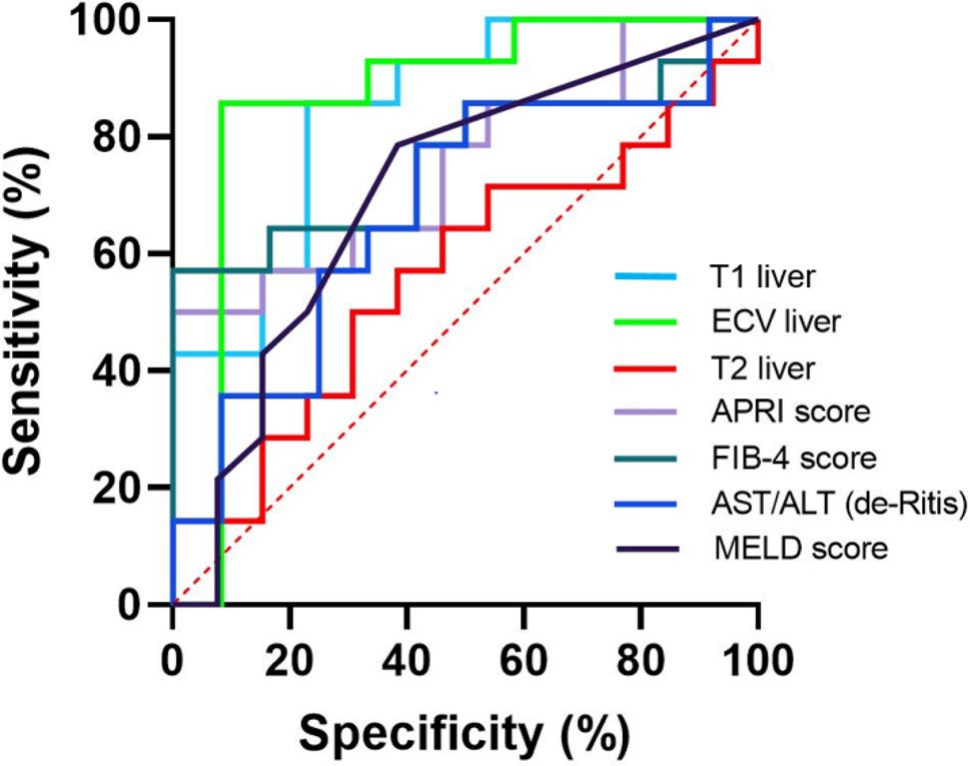
Graph show receiver-operating characteristic curves for diagnosis of significant fibrosis in patients with autoimmune hepatitis (≥ F2). Curves are given for hepatic T1 relaxation times (area under curve [AUC]: 0.846), hepatic ECV (AUC: 0.885), hepatic T2 relaxation times (AUC: 0.566), APRI (AUC: 0.852), FIB-4 score (0.758), ALT/AST ratio (de-Ritis) (AUC: 0.654), and MELD score (AUC: 0.654)

Several parametric mapping parameters were evaluated regarding the diagnostic performance to diagnose significant fibrosis (≥ F2). Regarding the overall diagnostic performance, hepatic ECV revealed the highest diagnostic performance with an AUC of 0.885, a sensitivity of 85.7%, and a specificity of 84.6% (cut-off value: > 29.5%). There were no significant differences in diagnostic performance of hepatic ECV and clinical fibrosis scores for diagnosing significant fibrosis: APRI (*P* = 0.702), FIB-4 (*P* = 0.138), and AST/ALR ratio (de-Ritis) (*P* = 0.058). Hepatic native T1 showed also a high diagnostic performance with an AUC of 0.846, a sensitivity of 85.7%, and a specificity of 76.9% to diagnose significant fibrosis (cut-off value: > 565 ms). There were no significant differences in diagnostic performance of hepatic ECV and native T1 (*P* = 0.550). Diagnostic performance of hepatic native T1 also differs not significant compared to the clinical fibrosis scores: APRI (*P* = 0.956) and FIB-4 (*P* = 0.346), AAR (*P* = 0.123). Diagnostic performance of hepatic T2 (AUC: 0.566) was significantly lower than that of hepatic native T1 (*P* = 0.006) and ECV (*P* = 0.004). Parameters of diagnostic performance for all other evaluated parameters with sensitivities, specificities, accuracies, positive and negative predictive values are given in Table 4. A ROC curves graph for diagnosis of significant fibrosis is given in Fig. 3.

**Table 4:** Diagnostic performance of different quantitative MRI parameters for assessment of MRE-derived liver stiffness in patients with autoimmune hepatitis and without (< F2) and with significant (≥ F2) fibrosis

Variable	AUC	Cutoff value	Sensitivity (%)	Specificity (%)	PPV (%)	NPV (%)	Accuracy (%)
Hepatic extracellular volume fraction (%)	0.885 (0.703–0.973)	> 29.5	85.7 (60.1–96.0)	84.6 (57.8–95.7)	85.7 (60.1–96.0)	84.6 (57.8–95.7)	85.2 (67.5–94.1)
Hepatic native T1 (ms)	0.846 (0.656–0.954)	> 565	85.7 (60.1–96.0)	76.9 (49.7–91.8)	85.7 (60.1–96.0)	76.9 (49.7–91.8)	81.5 (63.3–91.8)
Hepatic T2 (ms)	0.566 (0.363–0.754)	≤ 49.2	50.0 (26.8–73.2)	69.2 (42.4–87.3)	63.6 (35.4–84.8)	56.3 (33.2–76.9)	59.3 (40.7–75.5)
APRI score	0.852 (0.662–0.957)	> 0.521	85.7 (60.1–96.0)	76.9 (49.7–91.8)	80.0 (54.8–93.0)	83.3 (55.2–95.3)	81.5 (63.3–91.8)
FIB-4 score	0.758 (0.556–0.900)	> 2.055	57.1 (32.6–78.6)	92.3 (66.7–98.6)	61.5 (35.5–82.3)	85.7 (60.1–96.0)	74.1 (55.3–86.8)
ALT/AST ratio (de-Ritis)	0.654 (0.448–0.825)	≤ 0.976	78.6 (52.4–92.4)	53.8 (29.1–76.8)	64.7 (41.3–82.7)	70.0 (39.7–89.2)	66.7 (47.8–81.4)
MELD score	0.654 (0.448–0.825)	> 6	78.6 (52.4–92.4)	61.5 (35.5–82.3)	68.8 (44.4–85.8)	72.7 (43.4–90.3)	70.4 (51.5–84.1)

Data in parentheses are 95% confidence interval

*PPV* positive predictive value, *NPV* negative predictive value, *MELD* score model of end stage liver disease, *APRI* aspartate aminotransferase to platelet ratio index, *FIB-4* fibrosis-4-score; ASL/ALT ratio (de-Ritis): De-Ritis-Quotient

## Discussion

The purpose of our study was to evaluate the diagnostic utility of different quantitative parametric MRI parameters for the assessment of liver fibrosis using MRE-based liver stiffness as a reference standard in patients with AIH. The main findings of our study are that (1) hepatic ECV a native T1 showed a strong correlation with MRE-based liver stiffness and, (2) hepatic ECV and T1 showed a high diagnostic performance to diagnose significant fibrosis (≥ F2) in patients with AIH.

The development of liver fibrosis is a dynamic process characterized by the excessive extracellular matrix accumulation produced by fibrogenic cell populations in response to injury and inflammation. Advanced liver disease is characterized by increased production and decreased destruction of the extracellular matrix [35]. Consequently, an increased ECV leads to increased accumulation of extracellular MRI contrast agent in the extracellular space. Therefore, fibrosis is believed to increase the T1 relaxation time and ECV of the liver due to an increase in extracellular water and protein concentration*.* Moreover, recently published studies have already shown positive correlations between hepatic T1, T2, and ECV with liver fibrosis in both animal and human models [15, 18–20, 36]. There are also studies showing positive correlations between T1, T2 mapping parameters and MRE with liver fibrosis, however, without focusing on AIH patients [21, 37]. One of the most studied tools for non-invasive assessment of liver fibrosis in patients with AIH is liver stiffness measurement derived by TE (FibroScan) [38, 39]. Yet, it has limited diagnostic value and poor reproducibility and observer dependency, especially in patients with ascites and obesity [40]. There is one study, mentioning quantitative MRI techniques for prediction of portal hypertension in children and young adults with autoimmune liver disease [41]. There is still no literature directly showing correlations between MRE-based liver stiffness and MRI mapping parameter and its potential for detecting and staging liver fibrosis, focusing on patients with AIH.

Taken MRE-based liver stiffness as a reference standard, we found a strong correlation between hepatic ECV and liver stiffness (*r* = 0.80; *P* < 0.001) in patients with AIH. Furthermore, hepatic ECV showed a high diagnostic performance for detecting a significant fibrosis (F ≥ 2) in patients with AIH with an AUC of 0.885. Moreover, although it is not statistically significant, the diagnostic performance of hepatic ECV was higher to that of all non-invasive serologic tests. One drawback of the clinical scores is that fibrotic and inflammatory changes outside of the liver contribute to false positive results and, therefore, cannot be considered as liver specific. At the same time, quantitative mapping parameters, including ECV reflects directly the changes in the liver parenchyma itself. Furthermore, the use of ECV measurements seems to be beneficial, because on the one side, compared with conventional hepatic T1 and T2 mapping, it does not depend on parameters in image acquisition and the magnetic field strength. Therefore, ECV is physiologically normalized measure. Moreover, compared to MRE, its diagnostic quality might not be affected by obesity or ascites. Also, ECV calculation is possible on every MRI system, and no an additional expensive hardware is needed, which might be another advantage over MRE. The other mapping parameter, which showed high diagnostic performance in diagnosing significant fibrosis, is native hepatic T1 (AUC 0.846). Like ECV, T1 mapping seems to be more liver specific than laboratory markers as changes in hepatic T1 are measured directly in the liver parenchyma. In contrast to a previous study [21], which also showed positive correlation between MRE-based liver stiffness and hepatic T1 mapping (*r* = 0.49), our correlation was stronger (*r* = 0.69), likely because of the heterogeneous group of patients in the previous study with liver disease of different etiologies (including hepatitis B and C, non-alcoholic fatty liver disease, AIH, primary sclerosing cholangitis, and primary biliary cholangitis).

In contrast to previous data [21], we did not find significant correlation between T2 relaxation times and MRE-based liver stiffness in patients with AIH. Nevertheless, in the previous study, just poor to moderate correlation between T2 and T1 mapping as well as MRE was found. Generally, T2 mapping of the liver has not been validated in the clinical setting. Just a limited number of animal and human studies have shown that fibrosis can prolong the T2 relaxation time of the liver [21, 23]. It might be assumed that T2 relaxation time, similar to cardiac imaging, is might be increased in regions of fibrosis with coexistence of inflammation, due to increased water content [23]. Therefore, the absence of significant differences in both groups can be explained by the same inflammatory activity in the liver at the time of examination.

There are several limitations in this study. The main limitation was the absence of liver biopsy as a reference standard at the time of MRI examination. Liver biopsy with its clear drawbacks was performed only once for initial diagnosis. Liver biopsies for follow-up, however, are not routine clinical practice in our clinic, and therefore, ethics committee approval would have been unobtainable. Therefore, MRE-based liver stiffness was considered as a reference standard for the assessment of different liver fibrosis stages. T1 and T2 maps were acquired in a single transverse section at the level of the bifurcation of portal vein and, therefore, may have missed other significant changes, which probably occurred in other planes. Furthermore, our T1 measurements were not corrected for hepatic steatosis or hepatic/splenic iron overload, which might impair correct assessment of T1 values. However, there was no patient in our study collective with relevant steatosis as well as iron overload. Another limitation of our study was that the reading of all cases was performed only by one experienced radiologist. Additionally, the sample size was rather small and most patients in the advanced fibrosis group had F4 fibrosis, which might limit the overall applicability of our results. The study results have to be considered as preliminary, and further prospective studies using liver biopsy as the reference standard are necessary to confirm the accuracy and usefulness of ECV and other MRI parameters for assessment and follow-up of liver fibrosis in patients with AIH.

In conclusion, in our prospective study, we found strong correlations between quantitative hepatic MRI-derived mapping parameters including ECV and MRE-based liver stiffness. T1 mapping techniques with ECV calculation might provide additional diagnostic information over conventional MRI and over laboratory markers by non-invasive quantification and assessment of fibrotic liver changes in patients with AIH, without the need of additional equipment.

## Abbreviations

AIH: Autoimmune Hepatitis
MRE: Magnetic resonance elastography
ECV: Extracellular volume fraction
